# Environmental Insults to Glucose Metabolism: The Role of Pollutants in Insulin Resistance

**DOI:** 10.3390/ijms26188979

**Published:** 2025-09-15

**Authors:** Ewelina Młynarska, Mikołaj Grabarczyk, Klaudia Leszto, Gabriela Luba, Jakub Motor, Aleksandra Sosińska, Jacek Rysz, Beata Franczyk

**Affiliations:** 1 Department of Nephrocardiology, Medical University of Lodz, Ul. Zeromskiego 113, 90-549 Lodz, Poland; 2 Department of Nephrology, Hypertension and Family Medicine, Medical University of Lodz, Ul. Zeromskiego 113, 90-549 Lodz, Poland

**Keywords:** insulin resistance, glucose metabolism, environmental pollution, toxic metals, air pollution, pesticides, microplastics, nanoplastics

## Abstract

Insulin resistance is a condition of impaired tissue reactivity to insulin. This state is primarily associated with obesity and the lifestyle of modern Western societies, which favors abnormalities of glucose and lipid homeostasis. As a result, more and more people suffer from illnesses that develop because of the disturbed metabolic function of insulin, including type 2 diabetes, nonalcoholic fatty liver disease and polycystic ovarian syndrome. There are many studies describing the relationship between declining sensitivity to insulin and insufficient physical activity or unhealthy dietary habits. However, there is a vast number of other factors that may contribute to the development of this condition. In recent years, more attention has been paid to environmental pollutants as promoters of insulin resistance. As the overall grade of waste accumulation in the environment rises, factors like toxic metals, pesticides, dust, harmful gases and micro- or nanoplastics are starting to pose an increasingly serious threat in the context of metabolic disorder development. This review gathers data concerning the influence of the mentioned pollutants on the metabolic health of living organisms, with particular emphasis on the impact on carbohydrate processing, insulin resistance and molecular pathways associated with these processes.

## 1. Introduction

Insulin is a pancreatic hormone, with a peptide structure, responsible for the control of glucose metabolism. It is secreted by β-cells when serum glucose reaches a certain threshold [1,2]. The main target organs of its action include the liver, skeletal muscles and adipose tissue, where insulin stimulation induces glucose uptake and its storage in the form of glycogen and lipids. The state in which the mechanisms of responsiveness to physiological levels of insulin are impaired is described as insulin resistance [1,3]. This phenomenon lies at the basis of numerous diseases associated with a modern societies’ way of living that includes sedentary lifestyle, a substantial consumption of highly processed products and constant stress [4]. Many diseases encompass metabolic syndrome, such as type 2 diabetes (T2D), nonalcoholic fatty liver disease (NAFLD) and polycystic ovarian syndrome (POCS) [1,5]. On a molecular level, insulin resistance can be classified depending on whether it results from pre-receptor defects, receptor defects or post-receptor defects. Among pre-receptor defects, researchers mention mechanisms like genetically determined abnormalities of insulin particle structure, the incorrect or excessive degradation of insulin, the presence of antibodies specific to insulin molecules and overstimulation with other hormones that act as insulin antagonists—cortisol, glucagon, growth hormone or thyroxine [6]. Defects of an insulin receptor (IR) include its incorrect spatial structure, resulting in an impaired ability to bind insulin or trigger a biological response. A similar outcome may come from a decreased expression of its component peptides and consequently lower presence in cellular membranes [7]. The most complex set of insulin resistance causes comprises post-receptor anomalies. After binding an insulin particle, the IR goes through a conformational change that enables its rapid autophosphorylation and further recruitment of downstream signaling proteins, mainly insulin receptor substrates (IRSs), phosphatidylinositol 3-kinase (PI3K) and protein kinase B (PKB), also known as Akt. Dysfunction and abnormalities at any of these stages result in a downregulated metabolic response to insulin [8,9]. Moreover, post-receptor deficits also include the flawed functionality of transmembrane glucose transporters (GLUTs) and the inhibition of glycolysis, resulting from the excessive oxidation of fatty acids [6,7]. Other classification systems of insulin resistance focus more on the mechanisms that are most relevant for specific tissues. As mentioned above, the main effector organs for insulin action are skeletal muscles, the liver and adipose tissue. In terms of skeletal muscles, studies point particularly to defective GLUT4-dependent glucose transport, the impaired activity of the IR/IRS1/PI3K/Akt axis and unfavorably altered mitochondrial oxidative phosphorylation [1,10]. Hepatic insulin resistance manifests predominantly as increased gluconeogenesis and fasting hyperglycemia, primarily associated with the dysregulation of forkhead box protein O1 (FoxO1) transcription factor [1,11]. As for adipose tissue, the most important irregularities are excessive lipolysis and a constant inflammatory state, which impair insulin-dependent signaling [1,12]. There are many elements that contribute to the development of insulin resistance. Overweight, obesity, inadequate physical activity, genetic predisposition, chronic smoking, dyslipidemia, systemic inflammation, the abnormal secretion of adipokines and high sugar intake are just a few of a whole range of risk factors [12,13,14,15,16]. Furthermore, nowadays, more interest is being paid to agents such as medications, food additives and environmental pollutants as promoters of endocrine system dysfunction, insulin resistance and other metabolic disorders [17,18,19,20]. The World Health Organization (WHO) highlights indoor fuel combustion, second-hand tobacco use and ambient air pollution as the main sources of gaseous pollutants that cause disorders of glucose processing and consequently diabetes [21]. So far, more than 1000 complex compounds that meet endocrine disrupting chemical (EDC) criteria have been identified. The American Diabetes Association (ADA) underlines arsenic, persistent organic pollutants, phthalates and bisphenol as pollutants with the highest significance given to the induction of diabetes among exposed populations. By reaching the environment, with various kinds of waste, they alter adipose tissue differentiation, pancreas function or the neuroendocrine mechanisms of metabolism management and thus significantly promote insulin resistance [22,23]. A WHO statement, concerning health risks of various EDC, is consistent with these findings and marks fetal development, early childhood and puberty as critical and the most sensitive windows of exposure [24]. Apart from promoting unfavorable metabolic alterations and T2D, EDC can also have several negative consequences for neuropsychiatric health (autism, IQ loss, and attention-deficit hyperactivity disorder (ADHD)) and male and female reproductive systems (cryptorchidism, testosterone deficiency, and endometriosis) [25,26]. Recent analyses of the National Health and Nutrition Examination Survey (NHANES) showed that particular EDCs stimulate biological aging, with the activation of the receptor for advanced glycation end product (RAGE) pathway as the main underlying mechanism [27]. EDCs that favor weight gain are recognized as obesogens. Some of them are especially dangerous as certain evaluations reveal that they possess an ability to transmit the obese phenotype to subsequent generations not previously exposed to such compounds [28,29]. The complete set of substances that negatively affect the management of glucose metabolism and tissues’ reactivity to insulin is very wide and encompasses numerous air pollutants, toxic metals, plant protection products, elements of plastic waste and non-persistent metabolic disruptors like phenols, parabens, fluoroalkyles, phthalates or polycyclic aromatic hydrocarbons [17,30,31,32]. To provide an accurate and detailed, yet not verbose, review regarding the association of exposure to environmental pollutants and disruption of insulin signaling on molecular, histological and populational levels, the authors decided to focus on selected representatives of four groups of pollutants, air pollutants, pesticides, toxic metals and micro/nanoplastics, as they are referenced among highest ones of significance for the deterioration of human health in general [33].

## 2. The Role of Air Pollutants in Insulin Resistance

According to the World Health Organization (WHO), air pollution is a well-recognized risk factor for non-communicable diseases such as ischemic heart disease, stroke, chronic obstructive pulmonary disease (COPD), asthma, and cancer [34]. Emerging epidemiological evidence has also linked T2D and insulin resistance to particulate matter (PM) and other pollution exposure [35,36].

Air pollution is defined as the contamination of indoor or outdoor environments by chemical, physical, or biological agents that alter the natural characteristics of the atmosphere. The WHO’s data show that 99% of the global population breathes air that surpasses the WHO’s guideline limits and contains high levels of pollutants [34]. The main air pollutants include aerosols—dust, smoke, and fog—and gaseous substances like nitrogen dioxide (NO_2_), sulfur dioxide (SO_2_), benzene and ozone (O_3_). Aerosol pollutants can also be categorized by their particle size [37].

Population-level exposure to air pollutants has been increasingly characterized through large biomonitoring initiatives such as the European Human Biomonitoring Initiative (HBM4EU). This project provides harmonized data on internal exposure to various airborne pollutants, enabling comparisons across European populations. Although HBM4EU primarily focuses on exposure assessment rather than direct metabolic outcomes, these data complement epidemiological studies investigating the links between air pollution, oxidative stress, inflammation, and insulin resistance. Integrating exposure measurements with health outcomes is crucial for understanding how environmental pollutants contribute to metabolic disorders in European populations [38].

### 2.1. Particulate Matter (PM)

PM_2.5_ refers to fine particulate matter with a diameter ≤ 2.5 μm in contrast to PM_10_, which includes particles up to 10 μm. Due to its small size, PM_2.5_ remains suspended longer and penetrates deeper into the respiratory system and systemic circulation than PM_10_ [39]. PM composition includes transition metals (Fe, Cu, Zn, and Pb), polycyclic aromatic hydrocarbons (PAHs), and secondary particles formed by chemical reactions with gaseous compounds [40]. Cardiometabolic effects of PM_2.5_ are postulated to result from systemic inflammation, oxidative stress, and metabolic changes [40,41]. Inflammation in visceral adipose tissue, altered lipid metabolism in the liver, and impaired glucose consumption may play key roles in insulin resistance pathogenesis [35].

One of the first studies investigating metabolic changes related to ambient PM exposure revealed significant increases in the homeostatic model assessment of insulin resistance (HOMA-IR) after 9 days of PM_2.5_ exposure. In a randomized order, real air purifiers and sham purifiers (with the filter gauze removed) were placed in participants’ dormitories. After the exposure period, health tests were conducted: biological samples were collected within an hour, and the results were compared between participants with lower PM_2.5_ exposure (real purifiers) and those with higher exposure (sham purifiers). The results were also associated with elevated glucocorticoids, adrenocorticotropic hormone (ACTH), and corticotropin-releasing hormone (CRH), suggesting the activation of the hypothalamic–pituitary–adrenal axis [41].

To explore biological mechanisms of this phenomenon, the genome sequencing of 36 participants was conducted, revealing hypermethylation in genes linked to glucose and lipid metabolism, including *asxl2*, *lmna*, and *gnai3*. DNA methylation, particularly when it occurs in promoter regions of genes, is generally associated with gene silencing or reduced gene expression. Therefore, hypermethylation may lead to the downregulation of genes, potentially altering biological pathways relevant to the health effects of PM_2.5_ exposure [42]. *Asxl2* regulates glucose and lipid homeostasis via peroxisome proliferator-activated receptor gamma (PPAR-γ) pathway interactions [43]. Gene *lmna* influences lipid and glucose metabolism, with hypermethylation linked to insulin resistance in polycystic ovary syndrome patients [42,43]. *Gnai3* participates in inflammatory signaling pathways [42]. Other inflammation-related genes such as *prex1* (phosphatidylinositol-3,4,5-triphosphate dependent Rac exchange factor 1), *trim45* (tripartite motif containing 45), *rpap3* (RNA polymerase II associated protein 3), and *rap1gap2* (Rap1 GTPase activating protein 2) were also identified, indicating a broader epigenetic influence on immune response [42].

The meta-analysis conducted in 2018 assessed the association between exposure to ambient air pollutants and markers of insulin resistance. Their findings showed no significant association between PM_2.5_ exposure and insulin resistance markers, such as HOMA-IR and fasting insulin. However, they did observe significant positive associations for P10 and NO_2_, indicating that exposure to certain air pollutants—though not PM_2.5_ in this analysis—may contribute to impaired insulin sensitivity. However, the study has several important limitations that may explain the mixed or non-significant results for the association between PM_2.5_ exposure and insulin resistance. First, the number of included studies was limited, with substantial heterogeneity in exposure assessment methods and confounder adjustments, potentially masking true associations. Second, outcome measures relied solely on laboratory parameters, subject to measurement variability. Third, wide age ranges in study populations and a lack of data from highly polluted regions like China or India may have influenced the findings. Fourth, most studies applied single-pollutant models, failing to account for interactions between pollutants. Finally, the small sample sizes for PM_2.5_ and the use of random-effects models, which may underestimate statistical error, further weaken the strength of the conclusions. Together, these factors likely contributed to the heterogeneity and the absence of clear evidence linking PM_2.5_ exposure to insulin resistance in this meta-analysis [20].

Recent evidence also implicates gut microbiota as a mediator between PM_2.5_ exposure and insulin resistance. Zhao et al. has conducted a prospective panel study supporting the hypothesis that PM_2.5_ exposure may influence insulin resistance and type 2 diabetes risk by altering the gut microbiota and impacting serum metabolomic profiles. The findings suggest that PM_2.5_ is associated with increased insulin resistance markers and systemic inflammation and may disrupt sphingolipid metabolism—a key pathway in insulin resistance development. Moreover, changes in specific gut bacterial genera appear to partially mediate the link between PM_2.5_ exposure and sphingolipid metabolism, offering new insights into potential biological mechanisms connecting air pollution with metabolic disorders [44].

Emerging conceptual models, such as the OBS/REDOX framework, propose that environmental obesogens—including particulate air pollutants—disrupt metabolic signaling by inducing oxidative stress (ROS), mimicking hormonal triggers, and altering insulin secretion and energy balance. These pathways offer a strong mechanistic basis for how air pollution may promote insulin resistance beyond traditional inhalation-based effects [45].

### 2.2. Black Carbon (BC)

To evaluate the health effects of reducing household air pollution, a cookstove intervention trial was carried out in rural Intibucá, Honduras, where nearly 90% of homes rely on solid fuels and traditional wood-burning stoves. The study involved 230 participants, who initially used traditional stoves. In the subsequent stages of the study, participants received the Justa cookstove (equipped with a combustion chamber, chimney, griddle and side compartment to remove soot). At each stage of the study (which included both periods of using traditional stoves and periods using the Justa stoves), researchers collected finger-stick blood samples to measure HbA1c. In addition, 24 h personal and kitchen air pollution exposures were measured, focusing on PM_2.5_ and BC, the latter being a constituent of the PM_2.5_ fraction. The measurements were conducted as follows: personal exposure monitors were placed so that the air intake was positioned near the participant’s breathing zone. Kitchen exposure monitors were placed above the front edge of the primary cookstove. Later in the study, all personal exposure monitors for participants were replaced with the Ultrasonic Personal Aerosol Sampler (UPAS), which drew in air at 1.0 L per minute [46,47,48].

The intervention significantly reduced exposure to air pollution:

- Personal PM_2.5_ levels dropped by 48%: from a median of 82 μg/m^3^ (traditional stove) to 43 μg/m^3^ (Justa stove).
- Personal BC levels fell by 70%: from 11.8 μg/m^3^ to 3.5 μg/m^3^.
- Kitchen BC levels decreased by 86%: from 47.0 μg/m^3^ to 6.5 μg/m^3^.

These reductions were accompanied by modest improvements in metabolic health. The average decrease in HbA1c in the Justa group, compared to the traditional stove group, was −0.09 percentage points, a change comparable to effects seen in lifestyle interventions. Given the large global population exposed to household air pollution, even small improvements in HbA1c could translate into meaningful public health benefits [48].

### 2.3. Nitrogen Dioxide and Sulfur Dioxide

Nitrogen oxides, predominantly nitric oxide (NO), are produced by high-temperature combustion processes in vehicles and power plants. NO rapidly reacts with O_3_ to form NO_2_, a reddish-brown gas with a pungent odor. Indoor sources of NO_2_ include unventilated heaters and gas stoves [49]. Chronic exposure to NO_2_ has been associated with systemic inflammation and metabolic dysregulation, increasing the T2DM, as demonstrated in Indian and Chinese populations [50,51]. Inhaled NO_2_ induces reactive oxygen species (ROS) generation in the respiratory tract, triggering systemic oxidative stress and activating pro-inflammatory pathways such as nuclear factor kappa B (NF-κB). This leads to elevated circulating cytokines, including tumor necrosis factor α (TNF-α) and interleukin-6 (IL-6), which impair insulin signaling by promoting the serine phosphorylation of insulin receptor substrate (IRS) proteins, thereby disrupting glucose uptake. Additionally, NO_2_-induced endothelial dysfunction decreases NO bioavailability, further compromising insulin-mediated vasodilation and glucose delivery to peripheral tissues [50,51].

SO_2_ is a recognized air pollutant that contributes to environmental damage through acid rain formation and poses health risks by impairing respiratory function and lung health. Major anthropogenic sources of SO_2_ include mineral smelting, vehicle emissions, power generation, and various industrial processes [52,53]. Primarily linked to respiratory diseases, it also contributes to systemic inflammation and oxidative stress, potentially promoting metabolic dysregulation. Upon inhalation, SO_2_ dissolves in the respiratory tract lining fluid, forming reactive sulfite and bisulfite ions that facilitate ROS generation, causing oxidative damage to airway epithelial and systemic tissues [54]. This oxidative stress activates intracellular signaling pathways such as NF-κB and mitogen-activated protein kinases (MAPKs), enhancing the production of pro-inflammatory cytokines including IL-6 and TNF-α, which amplify inflammatory responses and tissue injury [54]. Moreover, SO_2_-induced endothelial dysfunction, characterized by reduced NO bioavailability and vascular inflammation, may impair insulin signaling and contribute to insulin resistance. However, epidemiological data directly linking SO_2_ exposure to insulin resistance remains limited and inconsistent [55].

### 2.4. Ozone

O_3_, a major component of photochemical smog, is formed by photochemical reactions between nitrogen oxides and volatile organic compounds under sunlight. As a highly reactive oxidant, ozone primarily affects the respiratory and cardiovascular systems but has also been implicated in metabolic dysfunction. Epidemiological studies suggest that chronic O_3_ exposure is associated with an increased risk of T2D and insulin resistance, particularly in urban populations with high ambient pollution levels [56,57]. Inhaled ozone induces the generation of ROS in airway epithelial cells and immune cells, initiating oxidative stress and systemic inflammatory responses. The activation of NF-κB and c-Jun N-terminal kinase (JNK) signaling pathways leads to the release of pro-inflammatory cytokines such as IL-6 and TNF-α, which impair insulin receptor signaling in peripheral tissues [57]. Additionally, O_3_ exposure has been linked to mitochondrial dysfunction, altered adipokine secretion, and endothelial dysfunction, all contributing to impaired glucose metabolism. An emerging mechanism is O_3_-induced telomere shortening, a marker of cellular aging and oxidative DNA damage. Shortened telomeres, observed in individuals exposed to high ambient O_3_, have been associated with increased systemic inflammation and insulin resistance, possibly due to premature cellular senescence and dysregulated metabolic homeostasis [56]. These findings highlight oxidative stress and accelerated cellular aging as potential mediators of O_3_-related metabolic risk, though further research is warranted to clarify causative pathways.

### 2.5. Benzene

To investigate the impact of benzene on glucose metabolism and energy homeostasis, researchers conducted a controlled inhalation study in mice. Animals were exposed to benzene at a concentration of 50 ppm for 6 h per day either for one day or over a period of up to four weeks. Blood glucose levels were continuously monitored using a real-time glucose tracking system [58]. Significant metabolic changes emerged early during exposure. By day four, mice exposed to benzene exhibited markedly elevated blood glucose levels (*p* < 0.001), with an approximately 20% increase observed by day seven compared to baseline (*p* < 0.01). These effects were specific to male mice and occurred without changes in body weight. To understand the underlying mechanisms, a transcriptomic analysis of the hypothalamus was performed 24 h after acute exposure. This revealed 405 upregulated and 332 downregulated genes in benzene-exposed mice. Gene ontology analysis identified insulin response as one of the most enriched pathways, with key insulin-related genes including *Sh2b2*, *Ogt*, *Klf15*, *Lpin2*, *Rab31*, *Ifg1r*, *Pik3ca*, and *Ptpn11* showing altered expression [59]. Functionally, benzene-disrupted insulin signaling in the hypothalamus. In control mice, insulin promoted the phosphorylation and cytoplasmic translocation of FoxO1, as well as the activation of pAkt and MAPK pathways. In contrast, benzene-exposed mice displayed elevated basal levels of pAkt and MAPK, which no longer responded to insulin stimulation. FoxO1 localization was similarly unresponsive, indicating hypothalamic insulin resistance. Further analysis of microglial cells via RNA sequencing revealed the activation of immune and NF-κB-related genes. The targeted deletion of inhibitor of nuclear factor kappa-B kinase subunit beta (I κ Kβ) in microglia or immune cells prevented benzene-induced gliosis, restored hypothalamic gene expression, and protected against hyperglycemia. This study confirmed that short-term benzene exposure rapidly induces hyperglycemia and disrupts central energy regulation in male mice. These effects are mediated through hypothalamic insulin resistance and microglial activation via the NF-κB pathway, highlighting a mechanistic link between airborne toxicants and the development of metabolic disease [58]. Human studies, including meta-analyses covering diverse populations such as children, adults, the elderly, and pregnant women, have shown a strong link between benzene exposure and insulin resistance. These analyses focused on urinary biomarkers, particularly trans,trans-muconic acid (t,t-MA) and reported adjusted odds ratios (ORs), related to metabolic disease risk. Exposure to benzene at levels typical of heavily polluted urban environments was significantly associated with an increased risk of metabolic disorders. The pooled analysis revealed a meta-OR of 1.47 (95% CI: 1.33–1.63, *p* < 0.001), providing robust evidence that environmental benzene exposure in humans is directly linked to impaired metabolic health [60,61,62].

### 2.6. Comparative Impact of Air Pollutants on Insulin Resistance

When comparing the harmful effects of various air pollutants on the development of insulin resistance, it is worth highlighting a systematic review and meta-analysis conducted by Gong et al. The study assessed the impact of PM_2.5_, PM_10_, NO_2_, and SO_2_ on insulin resistance, measured as adjusted percentage changes in the HOMA-IR values. The analysis revealed that for every 1 μg/m^3^ increase in pollutant concentration, PM_2.5_ was associated with a 0.40% change in HOMA-IR (95% CI: −0.03, 0.84; I^2^ = 67.4%, *p* = 0.009), while PM_10_ showed a stronger effect, inducing a 1.61% change (95% CI: 0.243, 2.968; I^2^ = 49.1%, *p* = 0.001). In contrast, NO_2_ had a much weaker association, with changes of 0.09% (95% CI: −0.01, 0.19; I^2^ = 83.2%, *p* = 0.002). These findings indicate that inhalable particulate matter, particularly PM_10_, exerts the greatest impact on insulin resistance compared to the other pollutants analyzed, as summarized in Table 1 [36].

To summarize the current understanding of how air pollutants may contribute to insulin resistance, Table 2 outlines the main proposed mechanisms identified in experimental and clinical studies.

It is worth noting that despite the growing body of the literature on the effects of air pollution on organisms and its influence on the development of insulin resistance, there is still a lack of conclusive evidence demonstrating increased susceptibility to these pollutants with advancing age. While some studies mention a higher proportion of insulin resistance development in older individuals, they do not exclude other confounding factors that contribute to insulin resistance progression with age per se. Moreover, the prevalence of metabolic diseases that independently promote insulin resistance increases exponentially with age [20,63]. Sex as a determinant factor influencing the variable intensity of pollution’s impact on insulin resistance development is also a subject of ongoing research. However, the data vary depending on reproductive age, the use or non-use of contraception, the coexistence of metabolic diseases, and smoking status. Therefore, this variable remains a topic for further investigation and represents, to some extent, a limitation in the conclusive information available on the subject [64,65].

**Table 2:** Overview of air pollutants and their proposed molecular mechanisms leading to insulin resistance.

Pollutant	Proposed Mechanisms of Insulin Resistance	Key References
PM_2.5_	- systemic inflammation and oxidative stress - visceral adipose tissue inflammation - altered lipid metabolism in liver - impaired glucose uptake - activation of HPA axis (↑ glucocorticoids) - epigenetic changes (hypermethylation of metabolic genes) - gut microbiota alterations (sphingolipid metabolism)	[20,41,42,44]
PM_10_	- similar to PM_2.5_ but less deeply penetrating	[20,36]
BC	- systemic inflammation linked to indoor air pollution - improvements in HbA1c with reduction in BC exposure - chronic low-grade inflammation and oxidative stress	[48]
NO_2_	- generation of ROS in respiratory tract - systemic oxidative stress and inflammation (↑ TNF-α, IL-6) - impaired IRS phosphorylation - endothelial dysfunction (↓ NO bioavailability)	[50,66]
SO_2_	- ROS formation upon inhalation (sulfite/bisulfite ions) - oxidative damage and systemic inflammation (↑ IL-6, TNF-α) - endothelial dysfunction - possible metabolic dysregulation (limited evidence)	[54,55]
O_3_	- ROS generation in airway and immune cells - systemic oxidative stress and inflammation (↑ IL-6, TNF-α) - mitochondrial dysfunction - altered adipokine secretion - endothelial dysfunction - telomere shortening linked to insulin resistance	[56,57]
Benzene	- central hypothalamic insulin resistance - disrupted insulin signaling (FoxO1, pAkt, MAPK pathways) - microglial activation via NF-κB - systemic inflammation and glucose dysregulation - human studies show strong association with insulin resistance	[58,60]

Abbreviations: PM_2.5_, fine particulate matter with a diameter ≤ 2.5 μm; PM_10_, fine particulate matter with a diameter up to 10 μm; BC, black carboon; HbA1c, glycated hemoglobin; NO_2_, nitrogen dioxide; ROS, reactive oxygen species; TNF-α, tumor necrosis factor alpha; IL-6, Interleukin-6; IRS, insulin receptor substrate; NO, nitric oxide; SO_2_, sulfur dioxide; O_3_, ozone; FoxO1, forkhead box protein O1; pAkt, phosphorylated protein kinase B (Akt); MAPK, mitogen-activated protein kinase; NF-κB, nuclear factor kappa-light-chain-enhancer of activated B cells. Notations: ↑ indicates increase/activation, ↓ indicates decrease/inhibition. Mechanistic findings summarize the effect of selected air pollutants on insulin resistance. Reported morphological, biochemical and molecular alterations include oxidative stress, inflammatory signaling, insulin signaling pathway components and lipid and glucose metabolism.

## 3. The Role of Pesticides in Insulin Resistance

Pesticides are natural or synthetic substances extensively used in agricultural production to protect crops from destruction. Based on their intended target, pesticides are classified into several categories, including insecticides, herbicides, fungicides and other, less commonly utilized groups. Alternative classification is based on chemical structure, distinguishing between inorganic and organic compounds, with the latter represented by the most frequently used subgroup of organophosphorous (OP) and organochlorine (OC) pesticides. Therefore, pesticides play a pivotal role in food production, significantly affecting crop yields [67,68,69]. Human pesticide exposure occurs via multiple routes, including inhalation, ingestion and dermal or ocular contact. Due to their lipophilicity, bioaccumulative and biomagnifying properties, pesticides accumulate particularly in adipose tissue, leading to long-term health effects [70,71]. Consequently, numerous studies have reported associations between pesticide exposure and the increased prevalence of various chronic diseases, including cancer, autoimmune diseases, reproductive, metabolic, cardiovascular, neurodegenerative, and respiratory disorders [68,71]. Several in vitro assessments have demonstrated the effects of specific pesticides on metabolic pathways, highlighting their role as obesogens and EDC [22,45]. Collectively, epidemiological and clinical evidence support a link between pesticide exposure, metabolic dysfunction, and insulin resistance. Experimental in vivo and in vitro studies are essential to elucidate how specific pesticides disrupt insulin signaling and glucose metabolism.

### 3.1. Pesticide Bioaccumulation and Markers of Insulin Resistance, Type 2 Diabetes and Metabolic Disorders

Children are particularly vulnerable to pesticide exposure, owing to higher intake relative to body weight, different metabolism, characteristic behavior (e.g., hand-to-mouth activity) and ongoing organ development. In addition, prenatal and breastfeeding exposures may adversely affect metabolic health in childhood [72,73]. A cohort study. reported that hexachlorobenzene (HCB) levels in cord blood were associated with child weight and BMI at 6.5 years. Moreover, the result was independent of maternal overweight, birth weight, sex and postnatal exposure to pesticides [74]. Additionally, another study showed that maternal serum concentrations of OC pesticides during pregnancy were associated with overweight or obesity among 9-year-old boys (118/261). The results concerning girls were not significant, suggesting possible sex-depending different effects after pesticides exposure [75].

The association between pesticide exposure and the development of T2D has been investigated in multiple studies. A meta-analysis included 17 cross-sectional studies and five prospective cohorts (follow-up range: 5–18 years). Elevated levels of chlorinated pesticides were correlated with higher fasting glucose, 2 h post-challenge glucose, fasting insulin, 2 h post-challenge insulin, and HOMA-IR values, although these associations did not reach statistical significance. Notably, a significant association between chlorinated pesticide exposure and T2D was observed in women, whereas data for men were insufficient to draw definitive conclusions [76]. Consistently, elevated OC pesticides levels were associated with high HOMA-IR among 749 nondiabetic participants with only background exposure to the pollutants. Moreover, among individuals with elevated levels of OC pesticides, waist circumference was strongly correlated with HOMA-IR [77]. As pesticides accumulate predominantly in adipose tissue, a study involving 50 participants measured concentrations of OCs in subcutaneous adipose tissue (SAT) and visceral adipose tissue (VAT). Analyzed pesticides included dichlorodiphenyltrichloroethanes (DDTs), chlordanes (CHLs), hexachlorocyclohexanes (HCHs), and HCB. Statistically significant associations with T2D were identified between DDT in VAT and CHLs in SAT. Furthermore, DDTs, CHLs and HCB concentrations were significantly associated with insulin resistance, irrespective of differences in absolute values between SAT and VAT [78]. Moreover, chronic inflammation is a well-established contributor to insulin resistance. In a cohort of 748 non-diabetic individuals, elevated serum concentrations of OCs were strongly associated with increased c-reactive protein (CRP) and HOMA-IR scores. Conversely, CRP was not associated with HOMA-IR among participants with low concentrations of OC pesticides [79]. A meta-analysis conducted by Lamat et al. comprising 12 studies with 6789 participants (29.1% with metabolic syndrome (MetS)) reported that pesticide exposure was associated with a 30% increased risk of MetS (OR = 1.30, 95% CI 1.22 to 1.37). Consistent with Song et al.’s findings, the association appeared to be sex-dependent, with men showing a relatively lower risk of MetS after pesticide exposure, despite a generally higher MetS prevalence among males. Moreover, a higher body mass index (BMI) and waist circumference were inversely associated with the risk of MetS, possibly reflecting pesticide accumulation in adipose tissue. This is further supported by studies indicating that increased amounts of pesticides are released into blood circulation after rapid weight loss [67,80]. Conversely, a cross-sectional study reported a lower prevalence of MetS among women in relation to urine glyphosate levels. Additionally, MetS incidence increased with age and was significantly associated with glyphosate exposure among older individuals (>65 years) [81]. Yet sex-dependent findings remain inconsistent and require further investigation. With increasing age, concentrations of OC pesticides have been shown to rise. However, glucose intolerance and insulin resistance may develop years after background environmental exposure to pesticides, particularly after the fifth decade of life, and independently of BMI [17,77,82]. Given the observed associations between pesticide exposure and disturbances in glucose metabolism, these compounds may serve as potential novel prognostic markers. In a prospective study, the metabolite 2-hydroxybiphenyl was significantly associated with the risk of developing T2D (133/431). However, additional studies are needed to confirm the causality of these findings [83]. As environmental exposure often involves mixed sets of different pollutants, rather than individual pesticides, many studies report the combined effects of multiple compounds. This reflects the biological outcomes of exposure to environmental pollution more realistically. However, it also makes an obstacle to elucidating health effects and molecular alterations exerted by specific pesticides. Therefore, examining isolated pollutants is essential to assess their direct impact on respective metabolic pathways [76,84].

### 3.2. Effects of Selected Pesticides on Glucose Metabolism Disturbances: Evidence from In Vivo and In Vitro Studies

The main findings concerning selected pesticides impact on glucose metabolism and insulin signaling are summarized in Table 3.

#### 3.2.1. Atrazine

Atrazine (ATR), a commonly used herbicide with a long half-life, poses a serious threat to living organisms’ health. ATR’s widespread utilization is associated with high risk of potential exposure for humans, as well as farm animals or pets. In adult Drosophila, long-term contact with ATR alone or combined with a carbohydrate-rich diet led to increased oxidative stress and favored the conversion of triglycerides into fatty acids due to a starvation response. Exposure to ATR induced hyperglycemia and increased Drosophila’s insulin-like receptor expression with the concomitant suppression of insulin-signaling. Combined with an excessive supply of carbohydrates, it showed a synergistic effect, promoting a T2D-like phenotype [85]. Additionally, ATR reduced the basal metabolic rate and suppressed insulin-mediated Akt phosphorylation, contributing to insulin resistance [86].

#### 3.2.2. Arsenite and Methylarsonite

In murine models, exposure to inorganic arsenic and its methylated metabolite methylarsonite disrupted hepatic glucose metabolism. Both compounds decreased hepatic glycogen synthesis by inhibiting the insulin-mediated activation of glycogen synthase (GS) and induced glycogen phosphorylase (GP). The regulation of GS and GP was mediated through the inhibition of the insulin-dependent phosphorylation of Akt [87].

#### 3.2.3. Permethrin

In male mice, co-exposure to permethrin and a high-fat diet significantly increased rodents’ weight gain and insulin resistance. Biochemical analyses revealed that TNF-α, a pro-inflammatory cytokine associated with obesity, was elevated. Moreover, GLUT4 expression was markedly reduced [88]. However, depending on the animal’s sex, effects exerted by permethrin lead to slightly different outcomes. In female mice, both high-fat diet and exposure to permethrin led to decreased voluntary activity. This pesticide also significantly suppressed insulin-stimulated glucose uptake through the downregulation of Akt, phosphoinositide-dependent kinase 1 (PDK1), and GLUT4. In contrast to the male model, high-fat diet and permethrin promoted the development of insulin resistance in females, but without a significant influence on the accompanying weight gain [88,89].

#### 3.2.4. Imidacloprid

Female mice simultaneously exposed to imidacloprid and a high-fat diet experienced significant weight gain due to alterations in lipid and glucose metabolism. Mice fed with imidacloprid presented an upregulated expression of cluster of differentiation 36 (CD36), sterol regulatory element-binding protein 1c (SREBP1c), and TNF-α, leading to the stimulation of fatty acid oxidation and inhibition of lipogenesis. Additionally, this pesticide increased the expression of phosphoenolpyruvate carboxykinase (PEPCK) and simultaneously decreased levels of peroxisome proliferator-activated receptor alpha (PPAR-α) transcripts. Such alterations indicated disrupted glucose and lipid metabolism [90].

#### 3.2.5. Lindane

In rat myoblast cultures, exposure to sub-toxic concentrations of lindane significantly inhibited insulin-stimulated glucose uptake. Total antioxidant levels decreased, while superoxide dismutase (SOD) activity increased. Moreover, lindane attenuated the insulin-dependent phosphorylation of IRS-1’s tyrosine and Akt’s serine in their respective active centers. Biochemical modulations also concerned other signaling proteins like inhibitor of nuclear factor kappa-B kinase subunit alpha (IκB-α), p38 MAPK and JNK, as the analyses revealed their increased phosphorylation. These changes, combined with the activation of heat shock protein 25 (HSP25) and induction of heat shock protein 70 (HSP70), suggest that lindane contributes to the promotion of insulin resistance mostly by exerting molecular alterations mediated by oxidative stress [91].

#### 3.2.6. Chlorpyrifos

In a study examining rat-derived myotubes, which were affected by sub-toxic doses of chlorpyrifos, the assessed cell cultures displayed significantly impaired insulin-stimulated glucose uptake. The researchers point to the disruption of intracellular signaling and altered activity of particular kinases as the main causes of these observations. Exposure to this pesticide leads to the suppressed phosphorylation of IRS-1’s tyrosine and Akt’s serine particles, with the coincident promotion of p38MAPK and IκBα phosphorylation [92]. The main findings concerning the selected pesticides’ impact on glucose metabolism and insulin signaling are summarized in Table 3.

## 4. The Role of Toxic Metals in Insulin Resistance

Environmental exposure to toxic metals has emerged as a significant factor in the pathogenesis of insulin resistance and T2D, driven by growing epidemiological evidence supporting their endocrine-disrupting properties [93]. Populations residing near industrial emission sources have been shown to exhibit increased diabetes risk, especially in association with air and waterborne toxic metal pollutants, suggesting a dose-dependent relationship between environmental contamination and glucose metabolism disturbances [93]. Likewise, data from large prospective cohorts and cross-sectional analyses consistently highlight the diabetogenic potential of certain metals, notably cadmium (Cd), lead (Pb), arsenic (As), mercury (Hg) and chromium (Cr), which may act through mechanisms involving oxidative stress, the dysregulation of gluconeogenesis, and interference with insulin signaling pathways [94,95,96]. While some metals such as zinc (Zn) and Cd appear to have dual or non-linear relationships with glycemic parameters depending on concentration and co-exposure patterns, others such as Pb and As have shown more consistent positive associations with elevated fasting glucose and diabetes incidence [95,96]. Notably, multi-metal exposures often exhibit interactive or synergistic effects, complicating the attribution of risk to single elements and underscoring the need for an integrated toxicological approach [93,94]. In the following sections, the pathophysiological and epidemiological links between Cd, Pb, As, Hg and Cr and their roles in glucose metabolism dysregulation will be discussed in detail, given their frequent identification as key contributors in both environmental and mechanistic studies. The main findings concerning toxic metals’ impact on the development of insulin resistance are summarized in Table 4. Across these metals, sex and reproductive status emerge as consistent effect modifiers of toxicokinetics and toxicity. Differences in absorption, tissue storage and mobilization, biotransformation, and interactions with estrogen/androgen signaling frequently alter both internal dose and biological response, implying that sex-stratified analyses and a consideration of reproductive stages (pregnancy, lactation, and menopause) are essential for accurate risk assessment [97,98,99,100,101,102,103,104,105,106,107,108,109,110,111].

### 4.1. Lead

Recent studies increasingly support the notion that environmental Pb exposure contributes to glucose metabolism impairment and may serve as a diabetogenic factor, particularly in vulnerable populations. A longitudinal analysis of over 5500 Chinese adults demonstrated that elevated blood lead levels were associated with higher fasting plasma glucose (FPG) and reduced β-cell function measured by homeostatic model assessment for beta-cell function (HOMA-B), especially among women, suggesting a sex-specific susceptibility to a lead-induced dysregulation of glucose homeostasis [112]. Sex-specific factors, including hormonal interactions with Pb, female-specific bone Pb mobilization during pregnancy/lactation/menopause, and differences in heritable versus environmental determinants of blood Pb, likely underlie the stronger associations observed in women and should guide stratified analyses in future studies [97,98,99,100,101,112,113,114,115,116,117]. This sex-differentiated effect may be partly attributed to enhanced oxidative stress responses in females, possibly mediated by interactions between Pb and estrogen, which amplify ROS generation and activate stress-sensitive pathways such as p38 MAPK, culminating in pancreatic β-cell apoptosis [113,114]. In support of these mechanisms, experimental data have demonstrated that Pb exposure disrupts insulin secretion and upregulates hepatic gluconeogenic enzymes, leading to hyperglycemia and insulin resistance [115,116,117].

To better contextualize these observations, it is important to consider sex-specific modifiers of lead kinetics and toxicity that may influence diabetic risk. Women may experience greater internal Pb burdens during life stages characterized by increased bone turnover (pregnancy, lactation, and the menopausal transition) because more than 90% of body lead is stored in bone and is mobilized along with calcium during these periods [97,98]. This endogenous mobilization can transiently raise circulating Pb levels during critical windows (fetal development, early childhood via maternal transfer, and postmenopausal bone loss), potentially exacerbating Pb-related oxidative and inflammatory insults to pancreatic β-cells and peripheral insulin signaling. Genetic and physiological determinants of Pb kinetics also show sex differences: twin studies indicate a substantial heritable component to blood lead in women (≈40%) versus predominantly environmental determinants in men (>95%) [99], which could modulate individual susceptibility to metabolic effects. Moreover, experimental and developmental immunotoxicity studies have reported more severe Pb-induced immune alterations in females following in utero exposure [100,101], providing a potential route by which sex modifies inflammatory mediators (e.g., high-mobility group box 1 (HMGB1), cytokines) that impair insulin action. Taken together, these factors imply that sex, reproductive history, and hormonal status should be considered as key effect modifiers in epidemiologic and mechanistic studies of Pb and glucose–insulin homeostasis.

In pediatric populations residing near e-waste recycling facilities, where ambient Pb exposure is chronically elevated, Zheng et al. observed that blood Pb concentrations positively correlated with fasting blood glucose and HOMA-IR indices, implicating Pb in early-life metabolic programming and systemic insulin resistance [118]. Interestingly, during the coronavirus disease 2019 (COVID-19) lockdown, when informal e-waste processing activities were significantly curtailed, the same group reported a concurrent reduction in children’s blood lead and PM_2.5_ exposure, accompanied by lower glycemic indices and improved insulin sensitivity, highlighting the reversible nature of environmentally driven metabolic alterations [118]. This phenomenon coincided with elevated serum levels of HMGB1, a pro-inflammatory alarmin associated with chronic low-grade inflammation and impaired insulin signaling via the toll-like receptor-4 (TLR4)/NF-κB and RAGE pathways, suggesting that inflammation may be a critical intermediary in Pb-induced metabolic dysfunction [119,120].

Further complicating the pathophysiological landscape, mixed metal exposure scenarios have revealed that Pb may act synergistically with trace elements such as Zn, potentially altering the directionality and magnitude of glycemic effects, though the interactive mechanisms remain poorly defined [94]. While some studies reported paradoxical inverse associations between Pb and glucose levels at specific exposure thresholds, these inconsistencies may reflect co-exposure to additional metals, non-linear dose–responses, or inadequate sample power [94]. Nonetheless, the preponderance of evidence implicates Pb as a persistent metabolic disruptor exerting its effects through oxidative stress, inflammatory signaling, and direct interference with insulin production and action, particularly in populations subjected to chronic low-dose exposure through air, water, and dust [95,115,118]. Importantly, sex-specific vulnerability appears to amplify Pb-related risks, highlighting the need for stratified analyses by sex and reproductive stage in future studies. These findings underscore the urgency of mitigating Pb pollution as part of broader public health strategies aimed at curbing the global rise in T2D.

Similarly to Pb, As also exerts its diabetogenic effects through oxidative stress and hormonal modulation, but differs in that its most significant exposure route remains contaminated drinking water.

### 4.2. Arsenic

Chronic environmental exposure to As, particularly through contaminated drinking water, has been increasingly implicated in the pathogenesis of T2D. Arsenic disrupts glucose metabolism through several converging mechanisms, including oxidative stress, apoptosis induction, mitochondrial dysfunction, and interference with insulin signaling pathways [121]. Arsenic trioxides (As^3+^) and arsenates (As^5+^) inhibit mitochondrial enzymes such as pyruvate dehydrogenase and α-ketoglutarate dehydrogenase, essential for oxidative phosphorylation, leading to lactate accumulation, ATP depletion, and energetic failure of metabolically active cells including pancreatic β-cells [122]. Furthermore, arsenic induces ROS, contributing to oxidative DNA damage and mitochondrial-mediated apoptosis, a process intensified by the upregulation of proapoptotic signaling cascades including the p53-cytochrome c-caspase axis [123].

As also affects insulin signaling directly by altering the expression of transcription factors such as pancreatic and duodenal homeobox 1 (PDX1), essential for insulin gene transcription, and PPAR-γ, which regulates adipocyte differentiation and insulin sensitivity. These effects are concentration- and time-dependent, with low-dose short-term exposure enhancing PPAR-γ expression, while prolonged or high-dose exposure inhibits adipogenesis and suppresses PPAR-γ activity [124]. Experimental models have also shown conflicting effects of arsenic on glucose uptake: while arsenic alone can enhance glucose transport in adipocytes, co-exposure with insulin attenuates this effect, suggesting a context-dependent impairment of insulin-mediated glucose utilization [125].

Epidemiological evidence further supports the diabetogenic role of arsenic. In a prospective analysis across two large U.S. cohorts—the Strong Heart Study and the Multi-Ethnic Study of Atherosclerosis—low to moderate levels of inorganic arsenic in drinking water (<10 µg/L) were significantly associated with increased T2D incidence, particularly in female participants and those with a BMI below 25 kg/m^2^, suggesting potential susceptibility in lean individuals. These associations persisted across urban and rural populations, even at arsenic concentrations well below the U.S. Environmental Protection Agency’s regulatory threshold, raising concerns about the sufficiency of current safety standards [126].

Genetic predisposition may further modulate individual vulnerability to arsenic-induced diabetes. A study conducted in arsenic-contaminated regions of Pakistan demonstrated a significant association between a single-nucleotide polymorphism (rs11191439) in the *AS3MT* gene, which encodes arsenic methyltransferase, and increased T2D prevalence, suggesting impaired arsenic biotransformation may exacerbate its metabolic toxicity. Elevated levels of arsenic were consistently detected in blood and urine samples of diabetic individuals compared to controls, corroborating the internal dose-dependent effect of As on glucose homeostasis [127]. Both mechanistic and population-level data converge on the conclusion that As acts as an endocrine-disrupting toxicant with a multifaceted impact on glucose metabolism. Its diabetogenic potential appears to be modulated by dose, exposure duration, genetic polymorphisms, co-exposures, and host metabolic phenotype. These findings underscore the urgent need for revisiting regulatory thresholds, improving exposure surveillance, and incorporating arsenic-induced metabolic dysregulation into public health risk assessments.

There are important sex-related differences that likely modulate arsenic’s effects on glucose–insulin homeostasis. Women tend to excrete slightly more dimethylarsinic acid (DMA) and less monomethylarsonic acid (MMA) than men [102], while the reduced intermediates MMA(III) and DMA(III) are highly toxic and may selectively damage pancreatic β-cells or disrupt insulin signaling [103]. Arsenic’s interactions with estrogen pathways, including effects on estrogen receptor α (ER-α) and estrogen-responsive gene expression observed in experimental models [104,105], may amplify or modify its metabolic impacts in females especially during pregnancy or other hormonally dynamic states. In males, arsenic retention in the reproductive tract and the arsenic-induced suppression of androgen production and spermatogenesis [106] can indirectly alter body composition and insulin sensitivity. Prenatal arsenic exposure also programs sex-specific long-term outcomes, producing divergent metabolic and gene-expression changes in male and female offspring [104,105]. Notably, sex modifies arsenic toxicity via differences in methylation profiles (MMA/DMA), hormone interactions (e.g., ER-α signaling) and prenatal programming effects, which help explain stronger or differential associations in women and support sex-stratified exposure assessment [102,103,104,105]. Taken together, these mechanistic and epidemiologic data indicate that studies of arsenic and glucose–insulin dysregulation should be stratified by sex and include an assessment of arsenic methylation profiles (MMA/DMA and their reduced forms), hormonal status (e.g., pregnancy, estrogen/androgen levels) and relevant genotypes (e.g., *AS3MT*) to accurately evaluate sex-dependent risk of insulin resistance and T2D. As in the case of Pb, sex differences substantially influence As’s diabetogenicity, with distinct methylation patterns, hormone interactions, and prenatal programming shaping susceptibility.

### 4.3. Cadmium

Cd, a pervasive environmental contaminant, has garnered increasing attention for its role in glucose metabolism impairment and its possible contribution to the etiology of T2D. Widespread dietary exposure, particularly through crops cultivated on contaminated soils, accounts for the majority of Cd intake in non-occupationally exposed populations, with cereals, potatoes, and vegetables constituting up to 80% of dietary Cd sources [128,129,130]. Once absorbed, Cd accumulates in various target tissues, including the pancreas and adipose tissue, where it can persist for decades and elicit biological effects that disrupt glucose homeostasis [131]. Women of reproductive age often have higher systemic Cd levels than men due to the increased intestinal uptake of Cd when iron stores are low, and these sex-related differences in uptake decline after menopause [97,107,108]. These sex-related differences in uptake and hormone-mediated immunotoxic responses indicate that reproductive stage (iron status, pregnancy, and menopause) and sex hormones should be incorporated as covariates or stratification variables when evaluating Cd-related metabolic risk [97,107,109].

Emerging evidence suggests that adipose tissue may serve as both a reservoir and a target of Cd toxicity. Cd accumulation in adipose tissue has been associated with higher insulin resistance and future risk of T2D, especially among smokers [132]. Given the endocrine function of adipose tissue in modulating energy balance and insulin sensitivity, Cd-mediated dysfunction in this compartment may represent a critical axis in the development of metabolic disease [133]. Moreover, Cd concentrations in adipose tissue were found to be higher and more variable among smokers, indicating a potential compounding effect of tobacco-derived Cd [134,135]. Cd exposure also initiates cellular processes that lead to inflammation and the generation of oxidative stress. In vitro and in vivo studies have demonstrated that Cd triggers the activation of Kupffer cells, neutrophil infiltration, and monocyte/macrophage-mediated secretion of pro-inflammatory cytokines such as TNF-α and IL-6, both of which are known to impair insulin signaling [136]. At micromolar concentrations, Cd increases the expression of inflammatory mediators including cyclooxygenase-2 (COX-2) and inducible nitric oxide synthase (iNOS), suggesting that even low-dose exposure may initiate chronic inflammatory responses implicated in metabolic dysregulation [137,138]. Concurrently, Cd activates NADPH and xanthine oxidases and uncouples endothelial nitric oxide synthase (eNOS), promoting excessive ROS generation, which damages cellular components and promotes β-cell apoptosis [139]. Experimental data indicate sex-specific immunotoxic effects of Cd and interactions with estrogen, implying that inflammatory and oxidative responses to Cd, and thus their impact on insulin signaling, can differ by sex and hormonal status [109].

A significant dose-dependent association between urinary Cd levels and elevated fasting plasma glucose or diagnosed diabetes was reported in NHANES III, even after adjustment for age, sex, ethnicity, and BMI [140]. Similarly, the Survey on Prevalence in East China for Metabolic Diseases and Risk Factors (SPECT)—China study linked elevated blood Cd levels with prediabetes, although not overt diabetes, highlighting the subtlety of metabolic changes at early stages of glycemic impairment [141]. Notably, cadmium-induced impairments in pancreatic β-cell function have been corroborated by postmortem data, showing age-related accumulation in β-cells [142], and by experimental studies revealing mitochondrial dysfunction, apoptosis, and suppressed insulin secretion in β-cells exposed to Cd [143,144]. Because sex hormones modulate β-cell function and mitochondrial resilience, Cd-induced β-cell injury and the capacity for recovery may vary between women and men and across life stages such as pregnancy and menopause [145].

Nevertheless, inconsistencies persist across epidemiological studies. While some prospective cohorts, such as those by Afridi et al. [146] and Little et al. [147], have reported higher Cd levels in individuals with diabetes, others, including Barregard et al. [148] and Borné et al. [149], have failed to detect significant associations. Discrepancies may stem from variations in population characteristics, exposure levels, smoking status, and the biological matrix used to assess Cd (e.g., blood vs. urine vs. adipose tissue), as each reflects different exposure windows [150].

Collectively, these findings support a multifactorial role of Cd in metabolic dysregulation. While not universally causal, Cd appears to contribute to insulin resistance and impaired glucose regulation through inflammatory, oxidative, and endocrine-disruptive mechanisms, particularly in vulnerable subpopulations and under conditions of chronic exposure. Further research employing uniform methods of exposure evaluation and long-term observational approaches are crucial to better understand the diabetogenic effects of Cd and its interplay with adjustable risk factors like dietary habits, smoking and adiposity. In summary, while epidemiological evidence remains somewhat inconsistent, Cd exposure is clearly capable of disrupting glucose homeostasis, with women of reproductive age appearing at heightened risk due to sex-related differences in absorption and immunotoxic responses.

**Table 4:** Diabetogenic mechanisms and epidemiological evidence of selected toxic metals.

Metal	Mechanisms of Action	Epidemiological Evidence	Key References
Pb	- Oxidative stress via ROS and MAPK activation - Disruption of insulin secretion and β-cell apoptosis - Upregulation of hepatic gluconeogenesis - Inflammatory response (HMGB1, TLR4/NF-κB, RAGE)	- Elevated blood Pb linked to ↑ FPG, ↓ HOMA-B, esp. in women - Children near e-waste sites had ↑ HOMA-IR - Sex-differentiated and reversible effects during COVID-19 lockdown	[112,115,116,118,119]
As	- Mitochondrial dysfunction, inhibition of PDH and α-KGDH - ROS-mediated DNA damage and apoptosis (p53-caspase axis) - Disruption of PDX1 and PPARγ activity - Impaired glucose uptake and adipogenesis	- Inorganic As (<10 µg/L) associated with ↑ T2D risk - Higher risk in lean females (BMI < 25) - SNP in AS3MT gene linked to T2D in As-contaminated areas	[121,122,123,125,126,127]
Cd	- ROS generation via NADPH/xanthine oxidase, eNOS uncoupling - Inflammation via TNF-α, IL-6, COX-2, iNOS - β-cell apoptosis and mitochondrial damage - Impaired insulin signaling and adipose tissue dysfunction	- Urinary Cd linked to ↑ FPG and prediabetes (NHANES III, SPECT-China) - High Cd in smokers’ adipose tissue - Mixed findings across prospective studies	[131,132,140,141,144,148]
Hg	- Disruption of redox balance and mitochondrial integrity - ↓ Insulin secretion, ↑ lipid peroxidation - Interference with PDX1, MafA, and IRS phosphorylation - Synergistic toxicity with dioxins	- High MeHg in seafood-eating populations linked to ↑ HOMA-IR - Autopsies from Minamata disease cases show β-cell damage - Dose-dependent associations observed in Asian cohorts	[151,152,153,154,155]
Cr	- Generation of oxidative stress and production of ROS - Induction of apoptosis in pancreatic β-cells - Mitochondrial dysfunction in insulin-sensitive tissues	- Meta-analysis of 15 RCTs (618 participants) found no association between Cr supplementation and glucose/insulin levels in non-diabetic subjects - Systematic review of 41 RCTs showed modest improvements in HbA1c (−0.6%) and FPG (−1.0 mmol/L) only in established T2D - Non-linear dose–response relationship observed between Cr dosage and body weight - Larger treatment effects predominantly seen in studies with methodological limitations - No consensus on reliable biomarkers for Cr status	[156,157,158,159,160,161,162]

Abbreviations: ROS, reactive oxygen species; MAPK, mitogen-activated protein kinase; FPG, fasting plasma glucose; HOMA-B, homeostasis model assessment of β-cell function; HOMA-IR, homeostasis model assessment of insulin resistance; PDH, pyruvate dehydrogenase; α-KGDH, α-ketoglutarate dehydrogenase; PDX1, pancreatic and duodenal homeobox 1; PPARγ, peroxisome proliferator-activated receptor gamma; SNP, single-nucleotide polymorphism; AS3MT, arsenic (+3 oxidation state) methyltransferase; TNF-α, tumor necrosis factor alpha; IL-6, interleukin-6; COX-2, cyclooxygenase-2; iNOS, inducible nitric oxide synthase; MeHg, methylmercury; IRS, insulin receptor substrate; Cr, chromium; Pb, lead; As, arsenic; Cd, cadmium; Hg, mercury; T2DM, type 2 diabetes mellitus; RCT, randomized controlled trial; HbA1c, glycated hemoglobin. Notation: ↑ indicates increase/activation, ↓ indicates decrease/inhibition. Epidemiological evidence summaries report associations (not necessarily causal relationships), strength and consistency vary by study design and population. Where sex differences are noted, they refer to subgroup analyses reported in the cited studies.

### 4.4. Mercury

Chronic exposure to Hg, particularly in its organic form methylmercury (MeHg), has been increasingly recognized as a contributor to glucose metabolism dysregulation and a potential risk factor for insulin resistance and T2D. MeHg, widely encountered through the dietary intake of seafood and rice, readily accumulates in metabolically active tissues, where it exerts toxic effects by disrupting redox balance, damaging mitochondrial integrity, and impairing insulin signaling pathways [151]. Experimental studies have demonstrated that exposure to MeHg leads to significant elevations in fasting glucose levels and HOMA-IR values, accompanied by reduced insulin secretion and β-cell dysfunction in animal models [152,153]. These effects were closely linked to the increased production of ROS and enhanced lipid peroxidation within pancreatic islets and insulin-sensitive tissues [152,154], with antioxidant co-treatment shown to mitigate these changes, highlighting the role of oxidative imbalance as a key intermediate.

Epidemiological evidence complements these findings. In a population-based cross-sectional study of 1449 non-diabetic Taiwanese residents from a historically mercury-contaminated area, higher blood Hg levels were independently associated with a substantially increased risk of insulin resistance, with individuals in the top exposure tertile exhibiting more than eleven-fold higher odds of elevated HOMA-IR compared to the lowest tertile after adjusting for relevant confounders. Additionally, autopsy data from individuals affected by Minamata disease—a condition caused by extreme MeHg exposure—revealed structural and functional damage to pancreatic islets, suggesting a link between Hg toxicity and β-cell impairment [155]. Although some studies report inconsistent associations between mercury biomarkers and T2D incidence, likely due to differences in exposure matrices, populations, or study design, pooled analyses indicate that Hg fulfills several criteria supporting a causative role, including strength, plausibility, coherence, and exposure–response trends [151].

Regional differences further shape the observed associations. Data from Asia, where dietary Hg exposure is often elevated, indicate stronger correlations with T2D risk, possibly due to higher MeHg burdens and co-exposure with other environmental contaminants [151]. For instance, Chang et al. reported that combined exposure to Hg and dioxins in seafood consumers significantly amplified the risk of insulin resistance compared to individual exposures, suggesting additive or interactive effects [155]. Moreover, Hg has been shown to interfere with key regulators of β-cell differentiation and function, such as PDX-1 and MafA, and to alter phosphorylation states of insulin receptor substrates, thereby impeding downstream insulin action [152,154].

There are sex-dependent factors that may modulate Hg’s effects on glucose–insulin homeostasis. Sex differences in MeHg metabolism, distribution and retention have been reported, with females showing faster whole-body clearance but higher peak kidney and brain percentages of MeHg and higher inorganic Hg in brain biopsies [110]. Females also exhibit greater mitochondrial antioxidant expression and higher brain catalase activity, while males may produce more nitrite/nitrate; estrogen provides additional antioxidant protection via ER-mediated pathways [111]. These metabolic and hormonal differences can modify pancreatic β-cell and insulin-sensitive tissue susceptibility to MeHg-induced oxidative stress and mitochondrial dysfunction, potentially yielding sex-specific risks for insulin resistance and T2D. Studies should therefore stratify by sex and consider exposure source, Hg species, antioxidant status, hormonal state (including pregnancy), and tissue retention patterns [110]. Consequently, sex differences in MeHg distribution, clearance, antioxidant capacity and estrogen-mediated protection necessitate sex-specific analyses and reporting to disentangle differential susceptibility to MeHg-induced insulin resistance [110,111].

These observations indicate that chronic low-to-moderate Hg exposure may contribute to the development of insulin resistance and T2D by disturbing glucose regulatory networks at multiple biological levels. Given the widespread presence of Hg in the global food supply and the growing burden of diabetes, greater attention to Hg as a modifiable environmental risk factor is warranted in both research and public health domains. Similarly to Pb, As, and Cd, MeHg’s diabetogenic effects are also modulated by sex-related differences in metabolism, antioxidant defenses, and hormonal status, suggesting that sex stratification is essential in evaluating risk.

### 4.5. Chromium

Cr occupies a unique position among metals affecting glucose metabolism due to its dual classification as both a purported essential micronutrient and a potential toxicant, contingent upon its oxidation state and exposure level. Unlike the unambiguously diabetogenic toxic metals previously discussed, Cr has generated considerable scientific interest for its hypothesized beneficial effects on insulin action and glucose homeostasis. Trivalent Cr (Cr^3+^) has been proposed to function as a component of the glucose tolerance factor and has been extensively marketed as a dietary supplement for glycemic control [156]. However, a critical evaluation of Cr’s efficacy through randomized controlled trials has produced inconsistent results, challenging earlier assumptions regarding its therapeutic value. Ali et al. conducted a methodologically rigorous randomized, double-blind, placebo-controlled trial investigating Cr picolinate supplementation (500 μg or 1000 μg daily for 6 months) in 59 adults with impaired fasting glucose, impaired glucose tolerance, or metabolic syndrome [156]. The researchers found no significant improvements in any primary outcome measures, including fasting plasma glucose, 2 h glucose during oral glucose tolerance testing, fasting or 2 h insulin levels, and a homeostasis model assessment of HOMA-IR compared to the placebo. Secondary outcomes, encompassing glycohemoglobin, anthropometric parameters, blood pressure, lipid profiles, and endothelial function, similarly showed no significant improvements with Cr supplementation [156].

These findings corroborate the results of a comprehensive meta-analysis by Althuis et al., which examined 15 randomized clinical trials involving 618 participants. This analysis demonstrated no association between Cr supplementation and glucose or insulin concentrations among non-diabetic subjects. While one large study of diabetic subjects in China reported beneficial effects, the pooled data from other studies involving diabetic participants failed to demonstrate significant benefits [157].

A subsequent systematic review by Balk et al. analyzed 41 randomized controlled trials and reported more nuanced outcomes. Among participants with established type 2 diabetes, Cr supplementation modestly improved glycosylated hemoglobin levels by −0.6% (95% CI −0.9 to −0.2) and fasting glucose by −1.0 mmol/L (−1.4 to −0.5) but showed no benefit in individuals without diabetes. The authors identified substantial limitations in the evidence base, including poor methodological quality in nearly half of the included trials, heterogeneity in study designs and outcomes, and inconsistent results. Notably, larger treatment effects were disproportionately observed in studies with methodological limitations, raising concerns about the reliability of positive findings [158].

The impact of Cr on body composition parameters in patients with T2D has also been investigated. Vajdi et al. conducted a meta-analysis of 14 randomized controlled trials and found that Cr supplementation did not significantly affect body weight (WMD: −0.26 kg, 95% CI: −0.69, 0.16), body mass index (WMD: 0.09 kg/m^2^, 95% CI: −0.03, 0.20), waist circumference (WMD: −0.47 cm, 95% CI: −1.10, 0.16), or fat mass (WMD = −0.43%; 95% CI −0.94, 0.09) in patients with type 2 diabetes [4]. Subgroup analysis revealed potential benefits in reducing fat mass specifically in older subjects (≥55 years) and when Cr picolinate was used as the intervention form. Additionally, a non-linear association was observed between Cr dosage and body weight, suggesting complex dose–response relationships [159].

The inconsistency in clinical outcomes may be attributed to several methodological and biological factors, including variations in baseline Cr status, differences in Cr formulations and dosages, heterogeneous study populations, and limitations in trial design [158]. The absence of consensus regarding reliable biomarkers for Cr status further complicates the interpretation of supplementation studies. Additionally, differences in insulin resistance severity among study populations may contribute to the variability in observed effects, with some researchers suggesting that only individuals with significant insulin resistance might demonstrate measurable responses to Cr supplementation [156]. This complex relationship between Cr and glucose metabolism underscores the importance of distinguishing between purported essential micronutrient functions and potential adverse effects when evaluating metals’ roles in metabolic disorders. While Cr continues to be widely marketed for glycemic control and weight management, current evidence does not provide strong support for its use in diabetes prevention or body composition improvement in non-deficient individuals, particularly those with prediabetes or at risk for T2D [156,157,158,159]. The case of Cr illustrates the necessity of rigorous scientific evaluation in determining the actual health impacts of metals on glucose metabolism, beyond their conventional categorization as either essential nutrients or environmental toxicants.

Practical implications: epidemiological and mechanistic studies should routinely (i) present sex-stratified results, (ii) adjust for or stratify by reproductive stage and relevant hormonal exposures (e.g., pregnancy, menopausal status, exogenous hormones), and (iii) include key biomarkers pertinent to each metal (e.g., bone Pb mobilization indicators, arsenic methylation indices MMA/DMA, Cd in adipose or urine) to improve causal inference.

Across Pb, As, Cd, and Hg, a recurring theme is that sex, hormonal status, and reproductive history strongly modulate toxicokinetics and diabetogenic potential. Differences in metal absorption, tissue distribution, biotransformation, and interactions with estrogen/androgen signaling consistently shape susceptibility patterns. This indicates that future studies on environmental metals and T2DM should stratify by sex, hormonal state (e.g., pregnancy, menopause), and relevant genotypes to accurately estimate risk.

## 5. The Role of Micro- and Nanoplastics in Insulin Resistance

### 5.1. Micro- and Nanoplastics Characteristics and Sources

Plastics are a class of materials with numerous desirable properties for various branches of industry. Depending on the basic polymer used for their production and the presence of possible side chains, they are classified as acrylics, polyesters, silicones, polyethylenes, polypropylenes, polyurethanes, and halogenated plastics [163]. Their relatively light weight, high durability and plasticity at the production stage make them an efficient element of different vehicles, electronics and clothing. Moreover, they are an indispensable element of everyday living, as they are often used as basic material to produce containers, disposable plates, bowls and cutlery. The estimated yearly production of plastics exceeds 400 million tons. More than 60% of this is plastic utilized for the composition of single-term use products, which are quickly turned into waste [164,165]. Different processes can lead to the disintegration of plastic deposits accumulated in the environment and eventually the formation of much smaller particles of micro- or nanometer size. Microplastics are described as plastic representatives with a particle size lower than 5000 μm. They can be classified as primary—originating from intentional production for specific purposes, mostly in the cosmetic industry—and secondary—originating from plastic waste though breakdown in physical, biological and chemical processes [165,166]. For particles with size < 1000 μm, the term “nanoplastics” is used [163]. In the present world, the overall pollution with plastic is so severe that microplastics are being detected in all kinds of environments and places, including air, water reservoirs and soil. As the presence of this pollutant is so widespread, it also easily penetrates its way into the food chains at various levels [163]. Most of the studies draw attention to the deposition of microplastics in seafood and salt extracted from sea water [167,168]. However, as it is common to store or prepare food in plastic containers, the processes that occur during food heating lead to the increased generation and precipitation of microplastic in all kinds of edibles [169,170]. A WHO report on potential human health implications of microplastics reveals that the concentration of this pollutant in both tap and bottled water can reach levels of even 10^4^ particles per liter. The same report states that when it comes to assessments of air, for the presence of plastic-derived pollution, the studies are inconsistent. However, the highest noted concentrations exceed 1500 particles per m^3^. Moreover, tire and road wear are marked as their main sources [171]. As a result, particles of micro- and nanoplastics can reach living organisms through various exposure routes and therefore affect the proper functioning of different tissues and metabolic processes. To this day, the consequences of carcinogenicity, pro-inflammatory influence, toxicity towards the reproductive system, the dysregulation of oxidation balance and gut dysbiosis have been reported [172]. Recently, more and more studies have been noting that exposure to micro- or nanoplastics can be associated with the disturbance of glucose processing and cause insulin resistance as well [173,174,175]. Although the set of potential nano- and microplastic particles is very wide and complex, depending on the specific source material, the topic of their association with insulin resistance development is still a fresh plane of research. This results in the fact that most of the available studies focus on polystyrene and polyvinyl chloride. Moreover, the vast majority are based on animal in vivo and in vitro models, lacking direct assessments of human subjects [171]. As the attainable data, discussed further in the next section of this review, indicates the presence of a serious threat, future examinations should also explore effects caused by other plastic-derived nano- and microparticles. Moreover, evaluations regarding effects of simultaneous exposure to multiple plastic pollutants would reflect real environmental conditions more precisely.

### 5.2. Micro- and Nanoplastics Influence on Glucose Homeostasis and Insulin Resistance

One of the first reports concerning microplastics effects on hormones associated with glucose homeostasis comes from a study performed on goldfish (*Carassius auratus*). The authors showed that exposure to the virgin polyvinyl chloride microplastics (PVC-MPs) is followed by a decrease in gene expression of growth hormone receptor (GHR) and insulin-like growth factor binding protein-1 (IGFBP-1). Simultaneously the increased transcription of mRNA for cortisol receptor (CR) was also noted [176]. Similar biochemical alterations in human subjects can be associated with central obesity and indicate rising hepatic insulin resistance [177]. Recent studies on zebrafish (*Danio rerio*) postulate that microplastic also affects expression of other genes that compose the GH/IGF axis, with IGFBP-2 and IGFBP-6 being downregulated [178,179]. Some alternative explanation for microplastic-caused insulin resistance might be the influence on mitochondrial function. Research focusing on the presence of nanopolystyrene in the environment found that concentrations of this pollutant, ranging between 1 and 100 μg/L, lead to induction of intestinal, mitochondrial unfolded protein response among larvae of *Caenorhabditis elegans*. Such response is associated with mitochondrial dysfunction [180]. Another study, utilizing a model based on human rhabdomyosarcoma cell line, provided further evidence for significance of mitochondrial damage in this matter, as polystyrene nano- and microplastics caused significant rise in intracellular Ca2+ levels and markedly diminished ATP production. Additionally, the insulin-dependent glucose uptake was impaired as well [181]. In the 3T3-L1 cell line, composed of adipocytes, the exposure to a mixture of nano- and microplastics induced increased adipo- and lipogenesis with the simultaneous deterioration of glucose influx, attributed to thediminished expression of GLUT4 [182]. Particles of nanopolysterene may also indirectly impair insulin-dependent signaling. Fan et al. found that ROS, the generation of which rises upon the administration of plastic nanoparticles, may trigger the phosphorylation of IRS-1. As a consequence, it leads to the disruption of PI3K/Akt signaling, a protein cascade linked with IR [183]. The mechanism responsible for such an outcome involves the ROS-mediated induction of inflammatory response by NF-κB signaling and activation of the antioxidant pathway through nuclear factor erythroid 2-related factor 2 (NRF2). This, in turn, contributes to the constitutive activation of MAPK, responsible for the stimulation of other downstream proteins and metabolic alterations [184]. Most noteworthy is the initiation of glucose-6-phosphatase (G6PC) and PEPCK genes transcription, as those are two crucial enzymes of the gluconeogenesis process [184]. Shi et al. point at the important role of microbiota disruption in the development of the polystyrene microplastic-driven induction of decreased hepatic sensitivity to insulin. The presence of micropolystyrene particles in drinking water administered to mice (*Mus musculus*) leads to an alteration of their intestinal microbiome and causes a perturbation in indole-3-carbaldehyde (I3A), phenylacetylglycine, betaine, choline, and carnitine metabolites levels. Each one of these compounds is treated as a biomarker of gut–liver axis disturbance leading to insulin resistance. Two weeks of exposure is enough to cause a detectable rise in fasting glucose and insulin levels and HOMA-IR values [174]. An even longer observation period, of 10 weeks, was adopted in a study by Huang et al. Here, impaired glucose tolerance among mice was also noted in an oral glucose tolerance test (OGTT) and insulin tolerance test (ITT). Moreover, liver tissue displayed a diminished expression of IRS-1, PI3K and PKB—major proteins of the insulin signaling pathway. The authors suggest that this might be the result of altered microbiota profile with an increased abundance of Bacteroidetes and decreased share of Firmicutes. The upregulation of pro-inflammatory factors like IL-6, IL-1β, TNF-α and CRP may also be of significance [185]. Similar results concerning impaired glucose homeostasis and the reduced overall diversity of bacteria inhabiting mice intestines were acquired by Zhao et al. However, microbiome analysis showed an increased abundance of Firmicutes instead of Bacteroidetes [186]. An important factor that furtherly exacerbates plastics’ negative influence on carbohydrate metabolism is a high-fat diet and the obesity that develops as its result. The mice that are exposed to micropolystyrene and excessive amounts of fat in their feeding chow demonstrate worse parameters of glucose tolerance and insulin resistance when compared with mice on a high-fat diet only [187]. Moreover, the continuous administration of microplastics significantly aggravates oxidative stress severity, which leads to the formation of lesions in the pancreas and liver—two organs crucial for the proper control of glucose metabolism [188]. The intensification of the observed alterations may also be due to potential modifications of plastic particles with different functional groups. Polystyrene nanoplastics with NH_2_ or COOH substituents display a stronger disrupting effect towards the correct management of glucose homeostasis than unmodified compounds. On a molecular level, this might be attributed to a more severe downregulation of pAkt and FoxO1 in liver cells [189]. Yang et al. showed that nanoplastics can affect an organism’s metabolic state also through air pollution. Mice exposed to nanoparticles of plastic in an atmospheric environment develop systemic inflammation and glucose intolerance as well as insulin resistance similarly to animals who ingest this kind of pollutant in their food [190]. Considering micro- and nanoplastics prevalence in the environment, potential interactions with other contaminants, food or medications may be of great importance. One pollutant with a confirmed exacerbating impact on plastic particles action is Pb. Co-exposure to Pb and polyvinyl chloride or polyethylene microplastics aggravates their undesirable metabolic effects. Moreover, it highly diminishes antioxidant enzyme activity in liver, precisely catalase (CAT), glutathione peroxidase (GPX) and SOD, furtherly worsening hepatocyte resistance to ROS [191]. Nanoplastics can bind with clarithromycin antibiotics and such cluster particles possess the ability to alter human insulin structure. As the study assessing this phenomenon was carried out in vitro, the exact consequences of such an influence are not clear, although the authors suggest that this may result in increased resistance to insulin actions because of its impaired biological function [192]. As the vast majority of currently accessible studies regarding the interplay between exposure to plastic particles and metabolic abnormalities or insulin resistance involve only cell cultures and animal models, it is impossible to predict the exact outcomes for human organisms. However, based on the available data, WHO experts emphasize that caution should be taken as the general degree of environmental pollution with plastic rises and so does the range of potential negative consequences for different health planes [171]. Evaluations based on data from the NHANES showed that exposure to nanoplastics, estimated by the intake of bottled water, correlates with a disruption of hepatic lipid metabolism and liver steatosis. Moreover, an increased risk of diabetes was associated with higher urine concentrations of different phthalates and organophosphate esters, which are common additives in plastic production. The referenced results are, however, only epidemiological and lack a direct assessment of molecular modulations and tissue damage in biological samples acquired from human subjects [193,194]. Future large-scale and long-term analyzes of plastic pollution and its metabolic outcomes for cohorts of human subjects could significantly improve our state of knowledge in this field. Moreover, possibly, they could reveal if nano- or microplastics pose an increased threat to various risk groups distinguished by sex, age, way of living or comorbidities. The micro- and nanoplastics effects on insulin resistance assessed in vivo studies are summarized in Table 5.

## 6. Future Directions and Translational Perspectives

### 6.1. Standardized Biomarkers of Environmental Exposure and Metabolic Effect

A reliable assessment of environmental insults requires standardized biomarkers that capture both exposure and metabolic consequences. Ryan et al. highlight that biomarkers are the preferred tools for linking environmental exposures to health outcomes. An ideal biomarker should reflect both the exposure and its metabolic effects, and the authors emphasize that using a combined panel of exposure, susceptibility, and effect biomarkers provides the most informative approach. This panel-based approach not only enhances early detection and comparability across studies but also strengthens causal inference by linking exposures directly to metabolic outcomes [195]. Molecular biomarkers can be broadly categorized into exposure and effect markers. Exposure biomarkers indicate prior contact with a chemical or environmental pollutant and are typically measured in bodily fluids or tissues. For example, in the context of glucose metabolism, serum levels of heavy metals or persistent organic pollutants serve as exposure biomarkers, reflecting the individual’s contact with insulin-resistance–promoting environmental insults. In contrast, effect biomarkers reflect the biological response to that exposure, signaling early metabolic changes or impairment. Markers such as HOMA-IR, fasting insulin, or HbA1c levels exemplify effect biomarkers in studies linking pollutants to insulin resistance, as they quantify the degree of metabolic disruption induced by environmental stressors [196]. Developing a consensus on these biomarkers will enhance comparability across studies and strengthen causal inference.

### 6.2. Exposomics for Stratifying Metabolic Risk

Exposomics provides a framework to capture high-dimensional environmental exposure data and identify individuals at greatest risk of metabolic disorders [197]. The external exposome can be assessed using personal sensors and wearables, geospatial models, and time–activity data, capturing real-world exposure patterns. The internal exposome reflects the biological response to these exposures and can be profiled via untargeted metabolomics, adductomics, non-targeted high-resolution mass spectrometry (HRMS), and an annotation of xenobiotic features [198,199]. By integrating exposomic data with genomic, epigenomic, and transcriptomic information, researchers can uncover gene–environment interactions and mechanistic pathways linking environmental insults to metabolic dysfunction [198,199]. Advanced analytical tools—such as Weighted Quantile Sum (WQS) regression, quantile g-computation, Bayesian Kernel Machine Regression (BKMR), causal inference approaches (directed acyclic graphs (DAGs) and negative controls), and machine learning risk scores validated against clamp or HOMA-IR—enable both risk stratification and mechanistic interpretation of complex mixtures [66]. Such integrative exposomic approaches are beginning to identify distinct high-risk subgroups, enabling precision prevention.

### 6.3. Preventive and Therapeutic Opportunities

Emerging strategies to mitigate pollutant-induced insulin resistance focus on microbiota modulation and targeted antioxidant therapies. Interventions such as probiotic supplementation or increased dietary fiber intake aim to restore gut microbial balance disrupted by environmental pollutants. Antioxidant approaches, including vitamins or NRF2 activators—compounds that stimulate the NRF2 pathway, a key defense mechanism that enhances antioxidant and detoxification responses. However, most of the current evidence is preliminary and requires further clinical validation [27]. Such interventions may provide accessible, low-risk strategies to counteract environmental insults on metabolism.

### 6.4. Regulatory and Public Health Implications

Translating mechanistic insights into policy and public health action is essential to reduce population-level risk. In clinical and public health practice, integrating brief environmental exposure history into metabolic risk assessment, offering guidance to high-risk groups (such as pregnant women, children, and workers), facilitating risk communication, promoting community-level exposure monitoring, and emphasizing environmental justice considerations are critical steps [200]. Evaluating the impact of these strategies requires tools like health-impact assessments that not only guide policy formation but also monitor health outcomes. The WHO defines HIA as “a practical approach used to systematically judge the potential health effects of a policy, strategy, plan, program or project on a population, particularly on vulnerable or disadvantaged groups” and notes it offers a framework for monitoring and evaluating changes in health as part of performance management [201]. A dual-track strategy—combining upstream regulation with downstream clinical screening as well as education and undergirded by evaluation metrics—offers a robust pathway to mitigate environmentally driven metabolic disease in both populations and individuals.

By combining standardized biomarkers, exposomic profiling, targeted preventive strategies, and regulatory measures, future research can transform mechanistic understanding into meaningful health improvements.

## 7. Conclusions

**Figure 1:**
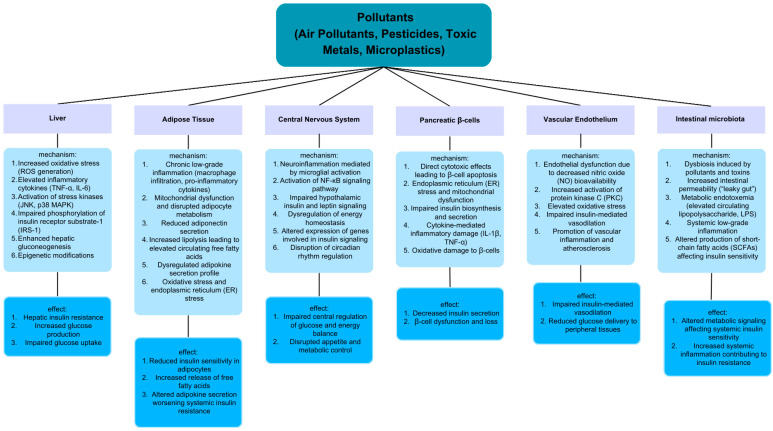
Summary of mechanisms and organ-specific effects of environmental pollutants driving the development of insulin resistance [20,202,203,204,205,206,207].

**Figure 2:**
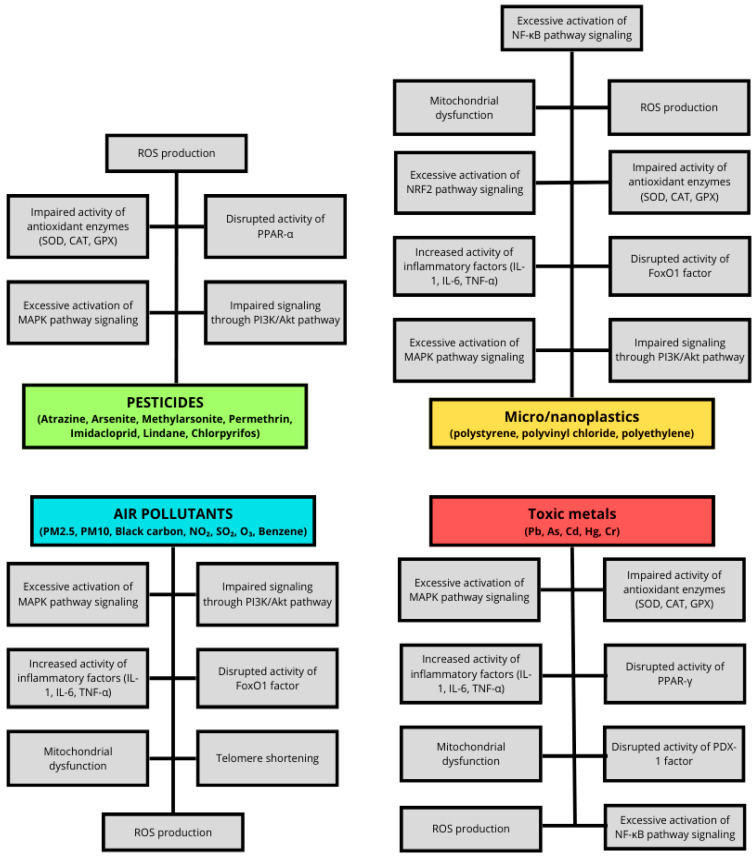
Summary of molecular mechanisms and alterations of signaling pathways associated with insulin resistance driven by environmental pollutants [20,112,115,116,118,119,121,122,123,125,126,127,131,132,140,141,144,148,151,152,153,154,155,156,157,158,159,160,161,162,171,202,203,204,205,206,207].

There is increasing recognition that environmental exposures play a meaningful role in the pathophysiology of insulin resistance. A growing body of evidence highlights the organ-specific and systemic impacts of air pollutants and other environmental toxins on metabolic regulation, which are summarized schematically in Figure 1. Effects displayed by various environmental pollutants on molecular mechanisms and signaling pathways associated with insulin resistance are summarized in Figure 2. Air pollutants such as PM_2.5_, NO_2_, ozone, and benzene contribute to metabolic dysfunction through a combination of systemic inflammation, oxidative stress, and disruptions in gut and epigenetic homeostasis. While individual associations, particularly with PM_2.5_, remain somewhat heterogeneous, broader patterns suggest that these exposures, especially in combination, pose a measurable risk to glucose regulation.

In parallel, the growing use of pesticides and the persistent presence of toxic metals in food, water, and air further compound the metabolic burden. Evidence links these substances to impaired insulin signaling, adipose tissue dysregulation, and direct β-cell toxicity. These effects are modulated by factors such as genetic predisposition, sex, and cumulative exposure, underscoring the complex nature of environmentally driven metabolic risk. Although research on micro- and nanoplastics is still in its early stages, the initial findings raise valid concerns. Their accumulation in biological systems appears to disrupt key molecular pathways relevant to insulin action, although robust data in human populations remain scarce.

Taken together, the literature points to a clear and actionable conclusion: environmental pollutants represent modifiable, and potentially preventable, contributors to insulin resistance. Their recognition as part of the broader metabolic risk landscape is essential for shaping future research agendas, public health strategies, and regulatory frameworks aimed at reducing the global burden of diabetes.

## Figures and Tables

**Table 1:** Comparative effects of selected air pollutants on insulin resistance (HOMA-IR) per 1 μg/m^3^ increase in concentration [36,55].

Pollutant	% Change in HOMA-IR (per 1 μg/m^3^)	95% CI	I^2^ (%)	*p*-Value
PM_2.5_	0.40%	−0.03 to 0.84	67.4	0.009
PM_10_	1.61%	0.243, 2.968	49.1	0.001
NO_2_	0.09%	−0.01, 0.19	83.2	0.002

Abbreviations: PM_2.5_, fine particulate matter with a diameter ≤ 2.5 μm; PM_10_, fine particulate matter with a diameter up to 10 μm; NO_2_, nitrogen dioxide; 95% CI, 95% Confidence Interval; I^2^, heterogeneity; HOMA-IR, homeostatic model assessment of insulin resistance.

**Table 3:** Summary of experimental evidence from in vivo and in vitro models showing the molecular effects on insulin signaling and glucose metabolism after exposure to selected pesticides.

Pesticide	Type of Study	Molecular Effect of Exposure to Pesticides	Key References
Atrazine	In vivo	↑ oxidative stress ↑ fatty acids ↓ triglycerides ↓ Akt	[85,86]
Arsenite and Methylarsonite	In vitro	↑ GS ↑ GP ↓ Akt	[87]
Permethrin	In vivo	↑ TNF-α ↓ GLUT4 ↓ Akt ↓ PDK1 ↓ GLUT4	[88,89]
Imidacloprid	In vivo	↑ CD36 ↑ SREBP1c ↑ TNF-α ↑ fatty acids ↑ PEPCK ↓ PPARα mRNA	[90]
Lindane	In vitro	↑ SOD ↑ IκBα ↑ p38 MAPK ↑ JNK ↑ HSP25 ↑ HSP70 ↓ antioxidant ↓ IRS-1 tyrosine ↓ Akt serine	[91]
Chlorpyrifos	In vitro	↑ p38MAPK ↑ IκBα ↓ IRS-1 tyrosine ↓ Akt serine	[92]

Abbreviations: Akt, protein kinase B; CD36, cluster of differentiation 36; GP, glycogen phosphorylase; GS, glycogen synthase; GLUT4, glucose transporter type 4; HSP25, heat shock protein 25; HSP70, heat shock protein 70; IκBα, inhibitor of nuclear factor κ-B kinase subunit α; IRS-1, insulin receptor substrate 1; JNK, c-Jun N-terminal kinase; PEPCK, phosphoenolpyruvate carboxykinase; PDK1, phosphoinositide-dependent kinase; PPAR-α, peroxisome proliferator-activated receptor α; p38 MAPK, p38 mitogen-activated protein kinase; SREBP1c, sterol regulatory element-binding protein 1c; SOD, superoxide dismutase; TNF-α, tumor necrosis factor α. Notations: ↑ indicates increase/activation, ↓ indicates decrease/inhibition. Mechanistic findings summarize the effect of selected pesticides on pathways associated with insulin resistance. Reported molecular alterations include oxidative stress, inflammatory signaling, insulin pathway components, lipid and glucose metabolism.

**Table 5:** Summarized results from in vivo studies assessing micro- and nanoplastics impact on insulin resistance and disturbance of glucose metabolism.

Plastic Type	Animal	Applied Dose/Concentration	Exposition	Results	Ref.
virgin polyvinyl chloride microplastics	goldfish	0.5 mg/L	4 days	↓ expression of GHR and IGFBP-1 ↑ expression of CR	[176]
micropolystyrene	zebrafish embryos	500 μg/L	30 days	↓ expression of GHR, IGF-1, IGFR-1, IGFBP-2 and IGFBP-6	[178]
nanopolystyrene	C. elegans	100 μg/L	6.5 days	↑ mitochondrial unfolded protein response in intestines	[180]
nanopolystyrene	mice	15 mg/kg (in feeding chow)	20 weeks	↑ plasma glucose levels ↑ ROS production ↑ phosphorylation of IRS-1 impaired PI3K/Akt pathway signaling	[183]
nanopolystyrene	mice	5 mg/kg (in feeding chow)	20 weeks	↑ ROS production ↑ activation of NRF2, NFκB and MAPK signaling pathways ↑ phosphorylation of IRS-1 ↓ Akt activity ↑ transcription of G6PC and PEPCK	[184]
micropolystyrene	mice	55 μg/day (in feeding chow)	2 weeks	↓ I3A and phenylacetylglycine generation ↑ 4-guanidinobutyric acid and CDP-choline generation ↑ fasting glucose and insulin levels ↑ HOMA-IR index value	[174]
micropolysterene	mice	80 mg/kg (in feeding chow)	10 weeks	↓ glucose tolerance ↑ HOMA-IR index value ↑ IL-1β, IL-6, TNF-α and CRP levels ↓ number of pancreatic islets ↑ vacuolization, nuclear pyknosis of hepatocytes ↓ microbiota richness ↑ *Bacteroidetes* presence in the intestines ↓ *Firmicutes* presence in the intestines	[185]
micropolystyrene	mice	1 μg/mL (in drinking water)	12 weeks	↑ body weight ↑ fat content ↑ fasting glucose ↓ glucose tolerance ↑ HOMA-IR index value ↑ HDL (high-density lipoprotein) ↑ expression of IL-6 and MCP-1 in perivascular adipose tissue	[186]
micropolystyrene	mice	0.125 µg/day (in feeding chow)	6 weeks	↑ blood glucose ↑ fasting insulin ↑ HOMA-IR index value	[187]
nanopolystyrene	mice	30 mg/kg (in feeding chow)	8 weeks	↑ blood glucose ↓ glucose tolerance ↑ insulin resistance ↑ ROS levels ↑ lesion formation in liver and pancreas	[188]
nanopolystyrene modified with –COOH and –NH_2_ functional groups	mice	5 mg/kg (in feeding chow)	9 weeks	↑ fasting blood glucose ↓ glucose tolerance ↑ HOMA-IR index value ↑ ROS levels in liver tissue ↑ glycogen accumulation in liver tissue ↑ cellular damage in liver and pancreas tissue ↓ p-Akt and FoxO1 levels in liver tissue All alterations were more severe in groups treated with nanopolystyrene modified with –NH_2_ functional groups	[189]
polyvinyl chloride and polyethylene microplastics combined with Pb	mice	10 mg/L (in drinking water)	6 weeks	↓ glucose tolerance ↑ glycosylation of serum proteins ↓ SOD, GPX and CAT activity in liver tissue ↓ HO-1 levels in liver tissue ↓ Keap1 and NRF2 mRNA expression in liver tissue	[191]

Abbreviations: CAT, catalase; CR, cortisol; CRP, C-reactive protein; GHR, growth hormone receptor; GPX, glutathione peroxidase; G6PC, glucose-6-phosphatase; HDL, high-density lipoprotein; HOMA-IR, homeostasis model assessment of insulin resistance; HO-1, heme oxygenase; IGF, insulin-like growth factor; IGFR, insulin-like growth factor receptor; IL, interleukin; IRS, insulin receptor substrate; I3A, indole-3-carboxyladehyde; MAPK, mitogen-activated protein kinase; MCP, monocyte chemoattractant protein; NF-kB, nuclear factor kappaB; NRF2, nuclear factor erythroid 2-related factor 2; PEPCK, phosphoenolpyruvate carboxykinase; PI3K, phosphatidylinositol 3-kinase; ROS, reactive oxygen species; SOD, superoxide dismutase; TNF-α, tumor necrosis factor α. Notation: ↑ indicates increase/activation, ↓ indicates decrease/inhibition. Mechanistic findings summarize the effect of selected micro/nanoplastics on various aspects of insulin resistance. Reported morphological, biochemical and molecular alterations include oxidative stress, inflammatory signaling, insulin signaling pathway components and lipid and glucose metabolism.

## Data Availability

The data used in this article were sourced from materials mentioned in References.

## Abbreviations

The following abbreviations are used in this manuscript:

ACTH	adrenocorticotropic hormone
ADHD	attention deficit hyperactivity disorder
As	arsenic
As^3+^	arsenic trioxide
As^5+^	arsenates
ATR	atrazine
BKMR	Bayesian kernel machine regression
BMI	body mass index
CAT	catalase
Cd	cadmium
CD36	cluster of differentiation 36
CHLs	chlordanes
COPD	chronic obstructive pulmonary disease
COVID-19	coronavirus disease 2019
COX-2	cyclooxygenase-2
CR	cortisol receptor
Cr	chromium
CRH	corticotropin-releasing hormone
CRP	c-reactive protein
DAG	directed acyclic graph
DDTs	dichlorodiphenyltrichloroethanes
DMA	dimethylarsinic acid
eNOS	endothelial nitric oxide synthase
ER-α	estrogen receptor α
FoxO1	forkhead box protein O1
FPG	fasting plasma glucose
G6PC	glucose-6-phosphatase
GHR	growth hormone receptor
GLUT	glucose transporter
GP	glycogen phosphorylase
GPX	glutathione peroxidase
GS	glycogen synthase
HBM4EU	European Human Biomonitoring Initiative
HCB	hexachlorobenzene
HCHs	hexachlorocyclohexanes
Hg	mercury
HMGB1	high-mobility group box 1
HOMA-B	homeostatic assessment for β-cell function
HOMA-IR	homeostasic model assessment of insulin resistance
HPA	hypothalamic–pituitary–adrenal
HRMS	High-resolution mass spectrometry
HSP	heat shock protein
I3A	indole-3-carbaldehyde
IGFBP-1	insulin-like growth factor binding protein-1
IκB-α	inhibitor of nuclear factor kappa-B kinase subunit α
IκK-β	inhibitor of nuclear factor kappa-B kinase subunit β
IL	interleukin
iNOS	inducible nitric oxide synthase
IR	insulin receptor
IRS	insulin receptor substrate
ITT	insulin tolerance test
JNK	c-Jun N-terminal kinase
MAPK	mitogen-activated protein kinase
MeHg	methylmercury
MetS	metabolic syndrome
MMA	monomethylarsinic acid
NAFLD	nonalcoholic fatty liver disease
NF-kB	nuclear factor kappaB
NHANES	National Health and Nutrition Examination Survey
NO_2_	nitric dioxide
NRF2	nuclear factor erythroid 2-related factor 2
OC	organochlorine
OGTT	oral glucose tolerance test
OP	organophosphorous
OR	odds ratio
PAHs	polycyclic aromatic hydrocarbons
Pb	lead
PDK-1	pyruvate dehydrogenase lipoamide kinase isozyme 1
PDX1	pancreatic and duodenal homeobox 1
PEPCK	phosphoenolpyruvate carboxykinase
PI3K	phosphatidylinositol 3-kinase
PKB	protein kinase B
PM	particulate matter
POCS	polycystic ovarian syndrome
PPAR-γ	peroxisome proliferator-activated receptor gamma
PVC-MPs	virgin polyvinyl chloride microplastics
RAGE	receptor for advanced glycation end-product
ROS	reactive oxygen species
SAT	subcutaneous adipose tissue
SO_2_	sulfur dioxide
SOD	superoxide dismutase
SPECT	Survey on Prevalence in East China for Metabolic Diseases and Risk Factors
SREBP1c	sterol regulatory element-binding protein 1c
t,t-MA	trans,trans-muconic acid
T2DM	type 2 diabetes mellitus
TLR-4	toll-like receptor 4
TNF-α	tumor necrosis factor α
UPAS	ultrasonic personal aerosol sampler
VAT	visceral adipose tissue
WHO	World Health Organization
Zn	zinc
